# Osteochondral autograft transplantation for malunited intra-articular fracture of the proximal interphalangeal joint: a case report

**DOI:** 10.1007/s00402-012-1622-4

**Published:** 2012-10-16

**Authors:** Nobuo Yamagami, Soichiro Yamamoto, Yumiko Tsujimoto, Yuji Uchio

**Affiliations:** 1 Department of Orthopaedic Surgery, Shimane University School of Medicine, 89-1 Enya-cho, Izumo, Shimane 693-8501 Japan; 2 Department of Orthopaedic Surgery, Wakakusa Daiichi Hospital, Osaka, Japan

**Keywords:** Proximal interphalangeal joint, Knee, Malunion, Osteochondral autograft transplantation

## Abstract

**Introduction:**

Malunited intra-articular fracture of the proximal inter-phalangeal (PIP) joint sometimes causes problems, such as range of motion (ROM) limitation in the joint or lack of digital dexterity; however, the treatment method has not yet been established. We report a juvenile case of osteochondral autograft tranplantation to treat a malunited intra-articular fracture of the middle finger.

**Case report:**

A 14-year-old boy was injured at the right middle finger by a baseball impact and underwent conservative treatment. At 5 months after the injury, he complained of continuing pain and restricted ROM. Plain X-ray and CT images showed a bony defect in the articular surface of the PIP joint of the right middle finger. He was diagnosed with malunited intra-articular fracture of the PIP joint and underwent surgical treatment. First, through a palmar incision, a columnar-shaped drill hole was made at the recipient site of osteochondral defect. Then a cylindrical osteochondral plug, 4.5 mm in diameter, harvested from the knee, was inserted into the recipient hole and press-fitted. One year after surgery, the patient has neither pain nor ROM limitation of the finger and the knee joint. MRI showed smooth articular surface of the PIP joint.

**Discussion:**

The benefits of our method include use of articular cartilage as a reconstruction material, availability for a relatively large cartilage defect, and stability of the autograft for the press-fitting method, which enable early mobilization exercise after surgery.

## Introduction

Malunited intra-articular fracture of the proximal inter-phalangeal (PIP) joint sometimes causes problems, such as range of motion (ROM) limitation in the joint or lack of digital dexterity. Recent treatment methods for reconstruction of articular cartilage disorder include tissue engineering and gene therapy as well as conventional arthrodesis, artificial joint replacement, and osteochondral transplantation. Of these, osteochondral autograft transplantation is believed to be the most reliable treatment method. Donor sites include the costo-osteochondral junction and the carpometacarpal (CM) joints [1–8], although a long-term prognosis is uncertain for the former due to the risk of ossification, while the latter is not sufficient to reconstruct a wide-range defect due to the limited size of osteochondral plug harvest. In our study, the donor site was selected from a non-weight-bearing portion of the lateral condyle of the knee joint, as it consists of an articular cartilage and relatively large osteochondral plug can be harvested. Herein, we report a case of osteochondral autograft transplantation (OAT) to treat a malunited intra-articular fracture of the proximal interphalangeal joint (PIP) of the right middle finger.

## Case report

Our case was a 14-year-old right-handed boy who complained of pain in his right middle finger. The finger was injured by a baseball impact, and the treating hospital performed splint fixation after diagnosing a fracture of the right middle finger. The patient failed to follow up his treatment on his own volition. However, pain in the right middle finger continued and the range of motion became restricted. He consulted our institution 5 months after injury.

**Fig. 1:**
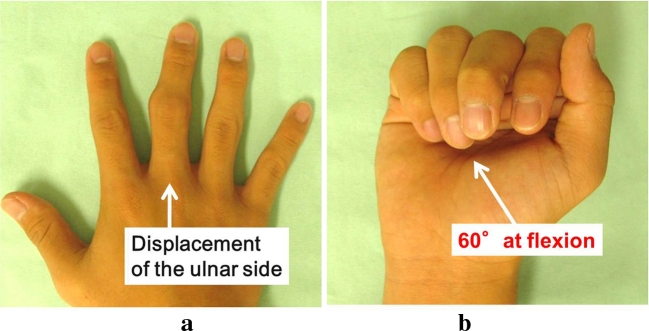
Photograph at the first consultation. **a** Displacement to the ulnar side is seen on extension. **b** The ROM of the PIP joint was restricted within 60° at flexion

At first consultation, there was swelling in the PIP joint of the right middle finger and displacement of that finger to the ulnar side at extension position (Fig. 1). The range of motion of the PIP joint was limited between extension 0° and flexion 60°.

**Fig. 2:**
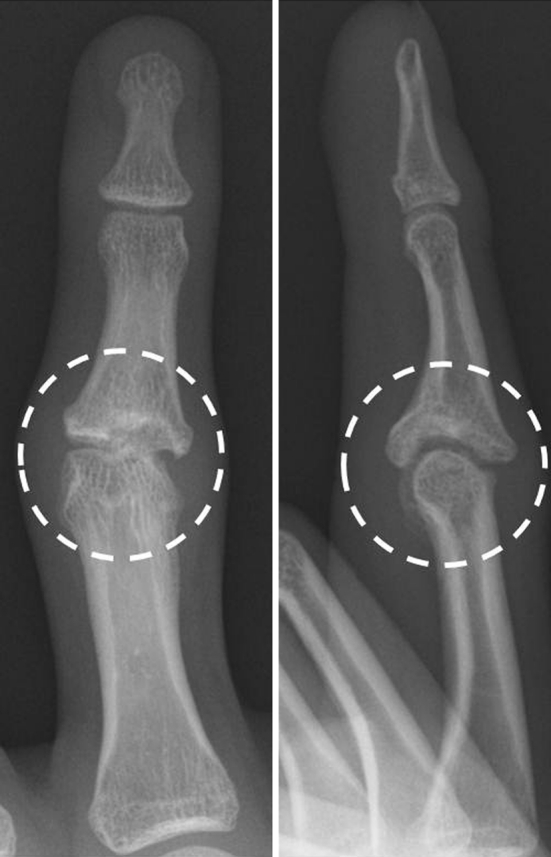
X-ray image before treatment. *Posterior* and *anterior view* show bone defect in the articular surface of the PIP joint of the middle phalanx and displacement of the middle finger to the ulnar side. *Lateral view* shows similar findings

**Fig. 3:**
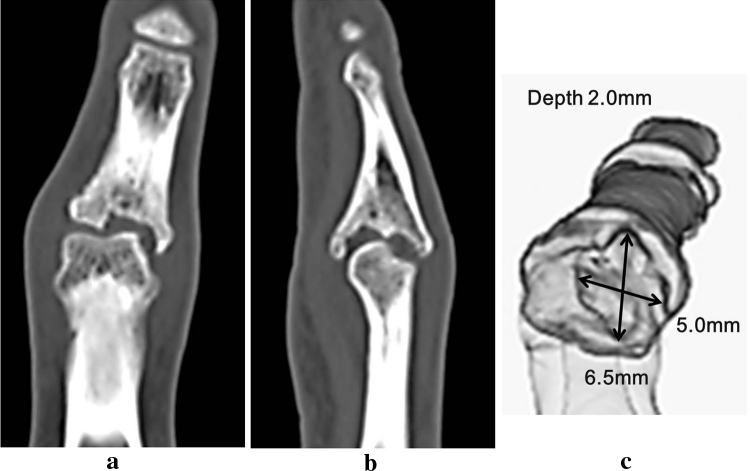
CT images before treatment. **a** Axial image, **b** sagittal image, and **c** 3D image. A bony defect 5.0 × 6.5 × 2.0 mm in size was seen in the articular surface of the PIP joint of the middle phalanx

**Fig. 4:**
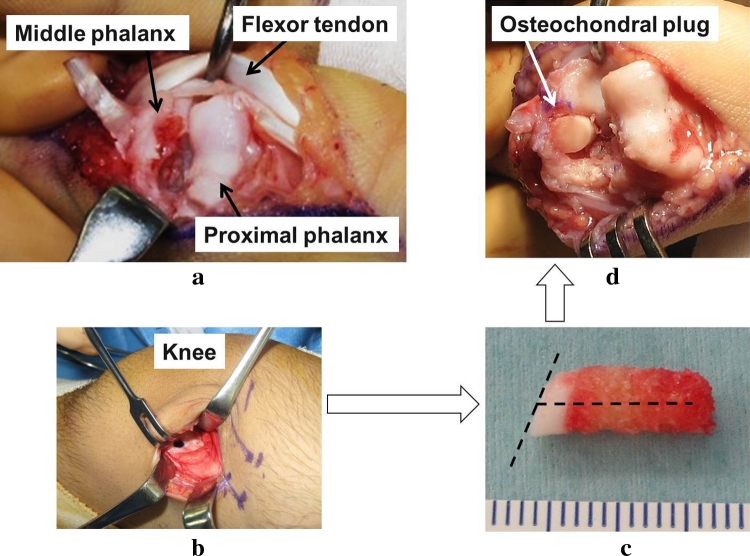
Operative procedure. **a** PIP joint was exposed by a palmar incision. Osteochondral defect of approximately 5 mm in diameter is seen in the articular surface of the middle phalanx. **b**, **c** Oblique osteochondral plug of 4.5 mm in diameter was harvested from the knee. **d** The drilled recipient hole was made in the osteochondral lesion of the middle phalanx, and the osteochondral plug was inserted and press-fitted

X-ray images of posterior and anterior views showed bony defect in the articular surface of the PIP joint in the middle phalanx and displacement of the finger to the ulnar side. X-ray image of the lateral view showed depressed articular surface of the PIP joint (Fig. 2). CT images showed a bony defect sized 5 × 6.5 × 2 mm in the articular surface of the PIP joint in the middle phalanx (Fig. 3). From these imaging findings, we diagnosed the case as malunited intra-articular fracture of the PIP joint and decided to conduct surgical treatment. First, an incision was made by palmar approach and the PIP joint was exposed. A cartilage defect approximately 5 mm in diameter was seen in the articular surface of the middle phalanx, and a cartilage defect of 1 × 2 mm in size was seen in the palmar side of the articular surface of the proximal phalanx (Fig. 4). After creating the drilled recipient hole at the osteochondral lesion of the middle phalanx, a cylindrical osteochondral plug of 4.5 mm diameter harvested from the left knee was inserted and press-fitted to the hole. The osteochondral plug was harvested using the mosaicplasty autogenous osteochondral grafting system (Acufex, Smith and Nephew, Andover, MA, USA) from a non-weight-bearing site on the upper lateral femoral condyle. The osteochondral plug was obtained with an obliquely angled cartilage surface along the long axis to facilitate insertion in the recipient hole (Fig. 4c). The cartilage defect in the proximal phalanx was left untreated as the range of damage was minimal.

Postoperative splint fixation was done only on the day of surgery, and mobilization exercise was started from the next day by changing the splint fixation to buddy taping. The buddy taping was continued up to 3 months after surgery. After removing the buddy taping, the patient gradually resumed sports activity.

**Fig. 5:**
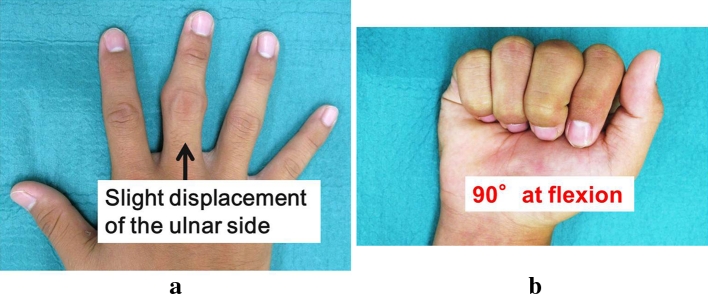
Photograph at 1 year after treatment. **a** Slight displacement of the PIP joint to the ulnar side remained. **b** The ROM of the PIP joint improved to 90° at flexion

**Fig. 6:**
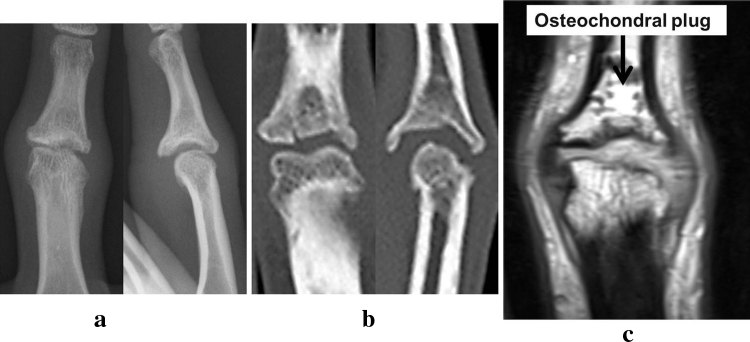
Imaging findings at 1 year after treatment. **a**, **b** X-ray and CT images show bone union with no dislocation of the osteochondral plug. Although slight displacement of the finger to ulnar side remained, the ulnar displacement of the axis improved from preoperative 14° to postoperative 8°. **c** MR image shows well-maintained joint space by the cartilage

As of 1 year after surgery, the patient has no pain, and the ROM of the PIP joint has improved showing extension and flexion to 0° and 90°, respectively. Although slight displacement to the ulnar side remains in the PIP joint, instability is not noted, (Fig. 5). There are no adverse effects in the donor site of the left knee. The patient resumed his previous level of baseball activity. Final follow-up X-ray and CT images showed bone union with no dislocation of the implanted osteochondral plug. Although slight displacement of the finger to ulnar side remained, the ulnar displacement of the axis improved from preoperative 14° to postoperative 8°. Also, MR images showed a well-maintained joint space by the transplanted cartilage (Fig. 6).

## Discussion

**Fig. 7:**
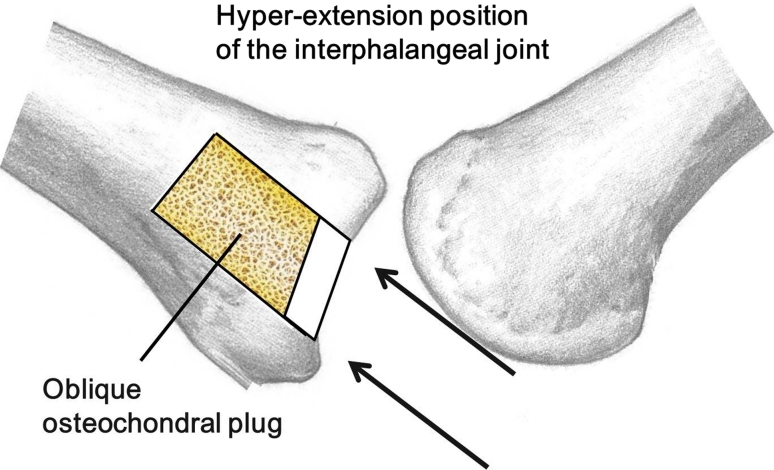
Schematic drawing of the osteochondral plug grafting. It can be applied to wide range defects and requires no internal fixation because of secure press-fitting. The plug can be inserted avoiding the head of phalanx because the osteochondral plug was obtained in an oblique shape

Reconstruction methods for the finger joints include perichondral graft, articular implantation with vessels, an artificial replacement arthroplasty of the finger, arthrodesis, and osteochondral autograft transplantation (OAT). Currently, OAT is considered the most reliable among those various methods with their respective benefits and drawbacks [1–8]. As a donor site for osteochondral autograft, the costo-osteochondral junction, and the carpometacarpal (CM) joints have been reported [1–8]. Osteochondral autografts from the knee have been used for surgical repair of osteochondral lesions in the knee, ankle, and elbow joint; however, no papers have reported the use of the application on osteochondral lesions of the finger joint. The costo-osteochondral junction can be used for a wide range of cartilage defects; however, manual trimming of the obtained osteochondral autograft is technically difficult and long-term treatment outcome is not clear due to the differing nature of the cartilage [1, 3, 4]. Grafting from the CM joint is beneficial to reconstruction by using articular cartilage and less invasiveness; however, as the amount of available cartilage is limited, it is not applicable for a wide range of defects [2, 5–8]. Our choice to harvest a graft from the knee joint was based on its component of articular cartilage, its usefulness in filling a wide-range of defects, and strong press-fit fixation, requiring no internal fixation (Fig. 7). Also, devices for harvesting osteochondral plugs from the knee joint are commercially available (OATS^®^ Arthrex Co., Naples, FL,USA) (MOSAICPLASTY^®^ Smith & Nephew Co., London, England), facilitating ease of operation. The demerits include the invasiveness to the unaffected knee and requirement for presence of cortical bone in the periphery of the recipient hole. However, no adverse effects of osteochondral graft harvest on donor knee function were reported after mosaicplasty for capitellar osteochondritis dissecans in young athletes [9]. Since our case was a juvenile patient, we considered articular cartilage to be the desired reconstruction material. We also needed a relatively large graft to fill the osteochondral defect which occupied more than half of the articular surface. Also, as the patient desired to resume sports activity as early as possible, the fixation of the graft should be firm and rehabilitation exercise should be started early after surgery. Considering these factors, we chose to perform OAT harvested from the knee joint.

There may be a concern of slight unevenness between the cartilage surface of the lateral condyle of femur (convexly curved) and the articular surface of the PIP joint of the middle finger (concavely curved). However, we believe the subtle difference will pose no problem because the finger joint is not a weight-bearing joint and a more important consideration is to eliminate any step-off of the cartilage surface. The cartilage of the femoral condyle is thicker than that of the finger joint; however, prevention of a step-off is possible by the deep insertion of the osteochondral plug. Trimming of the cartilage surface for prevention of a step-off is not recommended, as it may cause degeneration of the cartilage.

To insert the osteochondral plug, the head of proximal phalanx interferes with insertion of the osteochondral plug perpendicular to the joint surface. Miyamoto et al. [10] reported the oblique osteochondral plug transplantation technique for osteochondral autograft from the knee to treat osteochondritis dissecans of the elbow to enable insertion by avoiding the radial head. Utilizing this technique, in the present study, we obtained the osteochondral autograft obliquely and inserted the graft by avoiding the head of proximal phalanx at hyper-extension position of PIP joint (Fig. 7). We consider osteochondral autograft from the knee to be effective for treatment of malunited intra-articular fracture of the proximal interphalangeal joint.
